# A Novel Polyamine-Targeted Therapy for BRAF Mutant Melanoma Tumors

**DOI:** 10.3390/medsci6010003

**Published:** 2018-01-05

**Authors:** Molly C. Peters, Allyson Minton, Otto Phanstiel, Susan K. Gilmour

**Affiliations:** 1 Lankenau Institute for Medical Research, 100 Lancaster Avenue, Wynnewood, PA 19096, USA; PetersM@mlhs.org (M.C.P.); Mintonar@msn.com (A.M.); 2 Biomolecular Research Annex, University of Central Florida, 12722 Research Parkway, Orlando, FL 32826-3227, USA; Otto.Phanstiel@ucf.edu

**Keywords:** polyamines, α-difluoromethylornithine, polyamine transport system, melanoma, mutant BRAF

## Abstract

Mutant serine/threonine protein kinase B-Raf (BRAF) protein is expressed in over half of all melanoma tumors. Although BRAF inhibitors (BRAFi) elicit rapid anti-tumor responses in the majority of patients with mutant BRAF melanoma, the tumors inevitably relapse after a short time. We hypothesized that polyamines are essential for tumor survival in mutant BRAF melanomas. These tumors rely on both polyamine biosynthesis and an upregulated polyamine transport system (PTS) to maintain their high intracellular polyamine levels. We evaluated the effect of a novel arylpolyamine (AP) compound that is cytotoxic upon cellular entry via the increased PTS activity of melanoma cells with different *BRAF* mutational status. Mutant BRAF melanoma cells demonstrated greater PTS activity and increased sensitivity to AP compared to wild type BRAF (BRAF^WT^) melanoma cells. Treatment with an inhibitor of polyamine biosynthesis, α-difluoromethylornithine (DFMO), further upregulated PTS activity in mutant BRAF cells and increased their sensitivity to AP. Furthermore, viability assays of 3D spheroid cultures of mutant BRAF melanoma cells demonstrated greater resistance to the BRAFi, PLX4720, compared to 2D monolayer cultures. However, co-treatment with AP restored the sensitivity of melanoma spheroids to PLX4720. These data indicate that mutant BRAF melanoma cells are more dependent on the PTS compared to BRAF^WT^ melanoma cells, resulting in greater sensitivity to the PTS-targeted cytotoxic AP compound.

## 1. Introduction

Melanoma is a highly aggressive tumor with poor prognosis in the metastatic stage. Multiple oncogenic mutations (including genes encoding serine/threonine protein kinase B-Raf (BRAF)*,* the neuroblastoma RAS homolog (*NRAS*), and the proto-oncogene receptor tyrosine protein kinase KIT) drive this highly heterogeneous disease, with mutations in the *BRAF* gene detected in half of all melanoma tumors [1]. The treatment of metastatic melanoma has been revolutionized over the last decade with the discovery of highly prevalent *BRAF* mutations, which drive constitutive activation of the RAS-RAF-MEK-ERK pathway and promote uncontrolled proliferation [1]. Ninety percent of reported *BRAF* mutations result in substitution of glutamic acid for valine at amino acid 600 (the V600E mutation) [2,3]. The subsequent rapid development of selective inhibitors of mutant BRAF^V600E^ proteins (vemurafenib and dabrafenib) demonstrated a major advance in the treatment of melanoma patients harboring the BRAF^V600E^ mutation. However, nearly 100% of the patients exhibit disease progression within seven months after treatment with BRAF inhibitors [4,5,6]. Thus, new ways to overcome the acquired resistance to these inhibitors are urgently needed to increase survival in melanoma patients.

An alternative approach is to target a downstream pathway that is essential for survival of oncogene-addicted tumor cells. While oncogenes indeed drive proliferation, they do so via downstream effector molecules. For example, downstream of extracellular regulated kinase (ERK) signaling is c-MYC (myelocytomatosis viral proto-oncogene homolog), a known regulator of ornithine decarboxylase (ODC) transcription and polyamine biosynthesis [7]. The native polyamines (putrescine, spermidine and spermine) are amino acid-derived polycations that have been implicated in a wide array of biological processes, including cellular proliferation, differentiation, chromatin remodeling, hypusination of the eukaryotic initiation factor-5A (eIF-5A) and apoptosis [8]. Multiple oncogene-encoded proteins, including c-MYC and RAS, are known to upregulate key polyamine biosynthetic enzymes [7,9,10] as well as the cellular uptake of polyamines by activating the polyamine transport system (PTS) [11,12,13,14]. Compared to normal cells, tumor cells have been shown to contain elevated levels of polyamines [15,16,17,18]. These intracellular polyamine levels are maintained via tightly-regulated biosynthetic, catabolic, and uptake and export pathways [19]. Polyamine uptake is upregulated in many tumor types, especially in melanoma tumor cells when compared to normal cells [11,20]. Thus, melanoma tumor cells notoriously replete with multiple oncogenic mutations have a greatly increased need for polyamines compared to normal cells to meet their increased metabolic needs [20].

**Figure 1:**
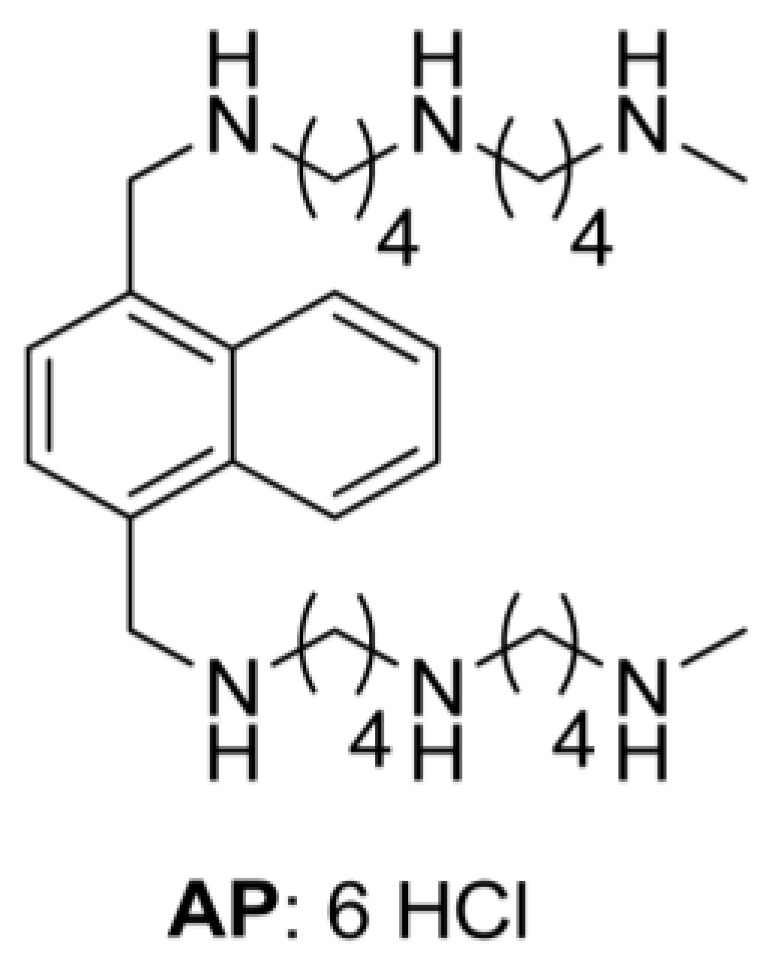
Structure of the arylpolyamine (AP).

Our objective was to exploit the oncogene-induced polyamine transport activity in melanoma cells by selectively targeting the PTS with a novel arylmethyl-polyamine (AP) compound (Figure 1, [21]). The two-armed design of AP predicated upon a naphthyl core provides PTS hyperselectivity and high potency [21]. Key to our drug design is that both exogenous polyamines and polyamine-based drugs are imported into tumors via a specific uptake system [8,21,22]. Here, we show that polyamine uptake is increased in mutant BRAF^V600E^ melanoma cells, and that AP treatment significantly increases cell death in BRAF^V600E^ melanoma cells compared to BRAF^WT^ melanoma cells. Furthermore, we show that BRAF inhibitor-resistance in melanoma tumor spheroid cultures can be overcome by treatment with AP. These studies provide valuable insights into developing more effective treatment strategies to restore sensitivity of melanoma tumor cells to BRAF inhibitors. In short, the mutant BRAF-driven polyamine addiction can be targeted by cytotoxic polyamine compounds, which selectively target melanoma cells with high polyamine import activity.

## 2. Materials and Methods

### 2.1. Cell Lines and Reagents

All human melanoma cell lines including WM983B, WM3734, WM3743, WM989, WM88, WM3451, WM3211, and 1205Lu were obtained as kind gifts from Dr. Meenhard Herlyn (The Wistar Institute, Philadelphia, PA, USA). These cells were maintained in MCDB153 (Sigma-Aldrich, St. Louis, MO, USA) and Leibovitz’s L-15 (Mediatech Inc, Manassas, VA, USA) medium (4:1 ratio) supplemented with 2% fetal calf serum and 2 mmol/L CaCl_2_. B16F10 cells were obtained from the American Type Culture Collection (Manassas, VA, USA) and maintained in Dulbecco’s Modified Eagle Medium (DMEM) (Invitrogen, Waltham, MA, USA) supplemented with 10% fetal bovine serum and 100 U/mL Penicillin/Streptomycin. The YUMM1.7 cell line (kindly provided by Marcus Bosenburg, Yale University, New Haven, CT, USA) that harbors a *BRAF^V600E^* mutation and inactivation of the Phosphatase and tensin homolog (*PTEN*) gene was maintained in DMEM/F12 (Invitrogen, Waltham, MA, USA) medium supplemented with 10% fetal bovine serum and 100 U/mL Penicillin/Streptomycin.

PLX4720, (S1152; Selleckchem, Houston, TX, USA) a derivative related to PLX4032/Vemurafenib (Plexxikon, Berkeley, CA, USA) was prepared as a 50 mM stock solution in dimethyl sulfoxide and stored at −20 °C. The synthesis of the AP compound has been described previously [21]. The compound was dissolved in phosphate buffered saline (PBS) to provide an initial stock (10 mM), which was filtered through a 0.2 um filter to ensure sterility. Subsequent dilutions were made in PBS to generate the desired stock solutions.

### 2.2. 3D Spheroid Culture

The nanoscale scaffolding NanoCulture plates (NCP) were purchased from (Organogenix Inc, Woburn, MA, USA). The base of each NCP is constructed with a transparent cyclo-olefin resinous sheet with a nanoscale indented pattern. To form spheroids, 1205Lu human melanoma cells were seeded in a 96-well NCP at 1 × 10^4^ cells/well in MCDB153 (Sigma-Aldrich, St. Louis, MO, USA) and Leibovitz’s L-15 (Mediatech Inc, Manassas, VA, USA) medium (4:1 ratio) supplemented with 2% heat-inactivated fetal calf serum and 2 mM CaCl_2_ and incubated in a conventional cell incubator at 37 °C in an atmosphere of 5% CO_2_ and normal O_2_ levels. When visible spheroids began to form on day 3 after the cells were seeded on the NCPs, treatment with PLX4720 and/or AP was initiated. After drug treatment for 48 h, the spheroid cultures were assayed for cell viability.

### 2.3. Cell Viability Assay

Cell proliferation assays were conducted in 96-well plates at 25–30% starting confluence to determine the effect of exposure to increasing concentrations of PLX4720 or AP with or without 1 mM α-difluoromethylornithine (DFMO) for 72 h. Cell viability was assessed using the EZQuant Cell Quantifying Kit (Alstem, Richmond, CA, USA) in which the tetrazolium salt WST-8 is reduced by the metabolic activity of live cells to formazan dye. For spheroids treated with PLX4720 and/or AP, viability of the spheroid cells was estimated by quantification of the adenosine triphosphate present using a CellTiter-Glo Luminescent Cell Viability Assay (Promega Co., Madison, WI, USA). The 72 h half maximal inhibitory concentration (IC50) values for AP were calculated using nonlinear regression (sigmoidal dose response) of the plot of percentage inhibition versus the log of inhibitor concentration in GraphPad Prism (v5; GraphPad Software, Inc., La Jolla, CA, USA). The IC50 value is defined as the concentration of the compound required to inhibit 50% cell viability compared to an untreated control.

### 2.4. Radiolabeled Spermidine Transport Assays

Polyamine transport in tumor cells was evaluated essentially as described previously [23,24]. Radioactive spermidine (Net-522, Spermidine Trihydrochloride, [Terminal Methylenes-^3^H(N)], specific activity 16.6 Ci/mmol; Perkin Elmer, Boston, MA, USA) was used. Cells were plated in 96 well plates and grown to approximately 80% confluence. Half of the cells were treated with 1 mM DFMO for 40 h. After repeated washing with PBS, ^3^H-spermidine was added at 0.5 µM and incubated for 60 min at 37 °C. Cells were then washed with cold PBS containing 50 µM spermidine and lysed in 0.1% sodium dodecyl sulfate solution at 37 °C for 30 min with mixing. Cell lysates were then aliquoted for scintillation counting and for protein assay using a microplate Bio-Rad protein assay (Bio-Rad, Hercules, CA, USA). Results were expressed as counts per minute (CPM)/µg protein.

### 2.5. Statistical Analysis

All in vitro experiments were performed at least in triplicate, and data were compiled from two to three separate experiments. Analyses were done using a one-way analysis of variance with a Tukey test for statistical significance or a Students *t*-test. In all cases, values of *p* ≤ 0.05 were regarded as being statistically significant.

## 3. Results

### 3.1. Human Mutant BRAF^V600E^ Melanoma Cells Are More Sensitive to Cytotoxic Effects of AP Than BRAF^WT^ Melanoma Cells

A panel of human melanoma cell lines with different BRAF mutational status was screened for their sensitivity to the BRAF inhibitor PLX4720. We confirmed previous findings that mutant BRAF^V600E^ melanoma cells, including WM983B, WM3734, 1205Lu, WM989, and WM88, demonstrated marked sensitivity to PLX4720 (IC50 values ≤ 3.0 µM), whereas BRAF^WT^ melanoma cells, including WM3451, WM3743, and WM3211, demonstrated relative resistance to treatment with PLX4720 (IC50 > 3.0 µM) [25]. This approach allowed us to rank the relative sensitivity of each cell line to the BRAF inhibitor, PLX4720. Thus, the sensitivity of these BRAF^V600E^ melanoma cells to BRAF inhibition with PLX4720 reflected their functional dependence on mutant BRAF signaling to sustain their proliferation and viability. 

**Figure 2:**
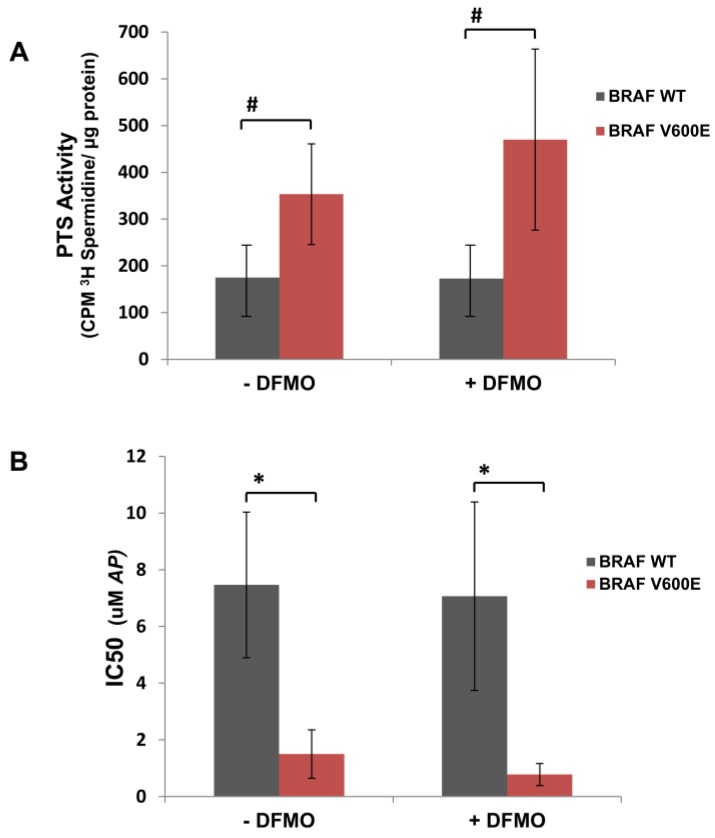
Greater PTS activity and increased sensitivity to AP in mutant BRAF^V600E^ human melanoma cells compared to wild type (WT) BRAF^WT^ cells. (**A**) BRAF^V600E^ human melanoma cells (WM983B, WM3734, 1205Lu, WM989, and WM88) and BRAF^WT^ human melanoma cells (WM3451, WM3743, and WM3211) were cultured with and without 1 mM DFMO for 40 h and then pulsed with 0.5 µM ^3^H-spermidine for 60 min at 37 °C. Cell lysates were assayed for CPM ^3^H-spermidine per mg protein by scintillation counting. The mean PTS activity ± SD for BRAF^WT^ melanoma cells is compared with that of BRAF^V600E^ melanoma cells under conditions where cells were cultured without added DFMO or with 1 mM DFMO. (**B**) BRAF^V600E^ human melanoma cells (WM983B, WM3734, 1205Lu, WM989, and WM88) and BRAF^WT^ human melanoma cells (WM3451, WM3743, and WM3211) were treated with increasing doses of AP with or without 1 mM DFMO, using 5–6 samples per dose of AP. After 72 h of culture, cell survival was determined via EZQuant Cell Quantifying assay (Alstem, Richmond, CA, USA). AP IC50 values were calculated by GraphPad Prism 6. The mean AP IC50 values ± SD for BRAF^WT^ melanoma cells is compared with that of BRAF^V600E^ melanoma cells under conditions where cells were cultured without added DFMO or with 1 mM DFMO; # *p* ≤ 0.05; * p < 0.01.

Likewise, we tested whether mutant BRAF^V600E^ cells were more sensitive to increasing concentrations of the cytotoxic polyamine transport ligand, AP (Figure 1), compared to BRAF^WT^ melanoma cells. Table 1 shows that mutant BRAF^V600E^ melanoma cells demonstrated greater sensitivity to AP (IC50 < 2.5 µM) than BRAF^WT^ melanoma cells (IC50 > 4.0 µM). This observation was reflected by the greater polyamine transport activity in BRAF^V600E^ melanoma cells compared to BRAF^WT^ melanoma cells (Figure 2A and Table 1). Since AP accumulated at a faster rate in BRAF^V600E^ human melanoma cells with higher polyamine transport rates compared to BRAF^WT^ human melanoma cells (Figure 2A), BRAF^V600E^ melanoma cells were significantly (*p* < 0.01) more sensitive to AP exposure than BRAF^WT^ melanoma cells (Figure 2B). In summary, cell lines with high polyamine import activity were more sensitive to the cytotoxic polyamine compound.

It is well known that lowering intracellular levels of polyamines with inhibitors of polyamine biosynthesis can increase uptake of extracellular polyamines as well as exogenous polyamine analogues [20,26]. WM983B and WM3743 melanoma cells were pretreated for 40 h with 1 mM DFMO, an inhibitor of ODC, the first and rate-limiting enzyme in polyamine biosynthesis, before measuring their polyamine transport activity. DFMO treatment dramatically increased polyamine transport activity in BRAF^V600E^ WM983B melanoma cells, but not in the BRAF^WT^ WM3743 melanoma cells (Supplementary Materials Figure S1A). In general, polyamine depletion with DFMO treatment enhanced polyamine uptake more in the screened human BRAF^V600E^ melanoma cells compared to that seen in BRAF^WT^ melanoma cells (Table 1, Figure 2A). Since DFMO treatment increases polyamine transport activity, we tested whether co-treatment with AP and DFMO will increase the sensitivity of melanoma cells to AP. For instance, BRAF^V600E^ WM983B melanoma cells are significantly (*p* ≤ 0.0001) more sensitive to AP treatment when co-treated with DFMO (IC50 = 0.7 µM) compared to that with AP alone (IC50 = 2.3 µM) (Supplementary Materials Figure S1C). In contrast, sensitivity to AP was not increased in DFMO-co-treated BRAF^WT^ melanoma cells (Table 1, Figure 2B). These data indicate that human BRAF^V600E^ melanoma cells demonstrate greater polyamine transport activity and increased sensitivity to AP compared to BRAF^WT^ melanoma cells, and their sensitivity can be increased by inhibition of polyamine biosynthesis with DFMO.

### 3.2. AP Is More Cytotoxic to BRAF^V600E^ Murine Melanoma Cells Than BRAF^WT^ Melanoma Cells

**Figure 3:**
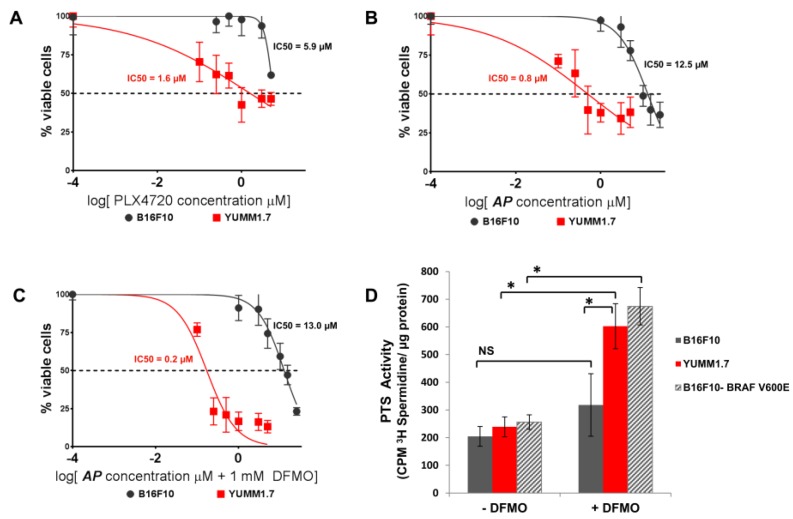
BRAF^V600E^ murine melanoma cells are more sensitive to AP than BRAF^WT^ melanoma cells. (**A**) Murine BRAF^V600E^ YUMM1.7 melanoma cells and BRAF^WT^ B16F10 melanoma cells were treated with increasing doses of PLX4720. After 72 h of culture, cell survival was determined via EZQuant Cell Quantifying assay. IC50 values were calculated by GraphPad Prism 6; *p* = 0.0013. (**B**) Murine BRAF^V600E^ YUMM1.7 melanoma cells and BRAF^WT^ B16F10 melanoma cells were treated with increasing doses of AP. After 72 h of culture, cell survival was determined via EZQuant Cell Quantifying assay. IC50 values were calculated by GraphPad Prism 6; *p* < 0.0001. (**C**) Murine BRAF^V600E^ YUMM1.7 melanoma cells and BRAF^WT^ B16F10 melanoma cells were treated with increasing doses of AP ± 1 mM DFMO. After 72 h of culture, cell survival was determined via EZQuant Cell Quantifying assay. IC50 values were calculated by GraphPad Prism 6; *p* < 0.0001. (**D**) YUMM1.7 and B16F10 melanoma cells and B16F10 cells retrovirally infected to express the mutant BRAF^V600E^ protein were cultured with and withoutS 1 mM DFMO for 40 h and then pulsed with 0.5 µM ^3^H-spermidine for 60 min at 37 °C. Cells were washed with cold PBS containing 50 µM spermidine, and cell lysates were assayed for CPM ^3^H-spermidine per mg protein by scintillation counting; * p < 0.0001; NS: not significant.

Since human melanoma cells possess multiple oncogenic mutations in addition to BRAF^V600E^, we compared AP cytotoxicity and PTS activity in the murine B16F10 melanoma cell line that is BRAF^WT^ with BRAF^V600E^ YUMM1.7 cell line that was derived from a melanoma tumor that spontaneously developed in a BRAF^V600E^/PTEN^null^ transgenic mouse [27]. As expected, YUMM1.7 cells were very sensitive to PLX4720 with a lower IC50 compared to B16F10 cells (Figure 3A). In addition, BRAF^V600E^ YUMM1.7 cells were significantly (*p* < 0.0001) more sensitive to AP and had a much lower IC50 value for AP compared to BRAF^WT^ B16F10 cells (Figure 3B). In particular, DFMO co-treatment increased the sensitivity of YUMM1.7 cells to AP (IC50 = 0.8 µM AP without DFMO and IC50 = 0.2 µM AP with DFMO co-treatment), and this correlated with a marked DFMO-induction of PTS activity in BRAF^V600E^ YUMM1.7 cells (Figure 3D). In contrast, BRAF^WT^ B16F10 cells demonstrated no significant induction in polyamine uptake following DFMO treatment (Figure 3D). However, B16F10 cells retrovirally infected to express the mutant BRAF^V600E^ protein exhibited a similar PTS activity profile as that seen with YUMM1.7 cells. DFMO treatment was shown to enhance polyamine uptake in the B16F10-BRAF^V600E^ cells as was seen with YUMM1.7 cells (Figure 3D). AP was also more cytotoxic in B16F10-BRAF^V600E^ cells (IC50 = 24.4 µM) compared to control-infected B16F10-pBABE cells that were infected with retrovirus expressing the empty plasmid (IC50 = 36.4 µM). These data suggest that melanoma cells with a mutant BRAF^V600E^ protein are more dependent on the polyamine uptake system compared to cells with a BRAF^WT^ protein, resulting in greater sensitivity to the PTS-targeted cytotoxic AP compound that enters and kills melanoma cells via the polyamine transport system.

### 3.3. Increased Resistance of Spheroid Melanoma Cells to PLX4720 Is Overcome with AP Co-Treatment

**Figure 4:**
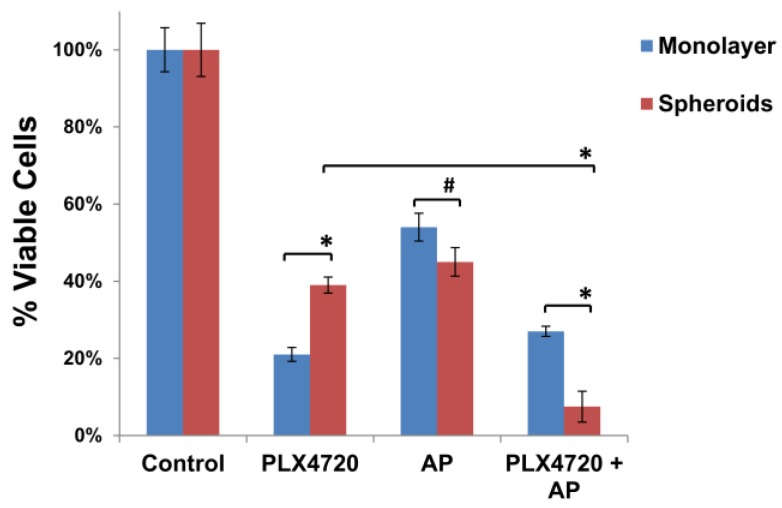
Increased resistance of spheroid melanoma cells to PLX4720 is overcome with AP co-treatment. BRAF^V600E^ mutant 1205Lu melanoma cells were seeded at 1 × 10^4^ cells in each well of 24-well NanoCulture plates (NCPs). When spheroids were formed on day 3, the 3D cultures of spheroids were treated with PLX4720 (25 µM) and/or AP (25 µM). 2D monolayer cultures of 1205Lu melanoma cells were also treated with PLX4720 (25 µM) and/or AP (25 µM). After drug treatment for 48 h, the viability of spheroids and monolayer cultures was assayed using the CellTiter-Glo Luminescent Cell Viability Assay. The percent cell survival in each treatment group was calculated relative to cells treated with medium only under the same conditions. As controls, the growth of cells without drug treatment under each condition was normalized as 100% separately. The means are presented ± SD; * p < 0.0001; # *p* = 0.0028.

Because growth of cells in a 3D culture system has been found to be more representative of the in vivo microenvironment, we cultured 1205Lu human melanoma cells using a nanoscale, scaffold-based NCP in which tumor cells easily form 3D spheroids [28]. Although these tumor spheroid cultures are grown in ambient air, the spheroid microenvironment closely resembles that in tumors with a hypoxic core and is more relevant for drug sensitivity compared to that seen with monolayer cultures [28,29,30]. Similar to previous reports [31], BRAF^V600E^ mutant 1205Lu melanoma cells grown as spheroids on NCPs were more resistant to 48 h treatment with PLX4720 (25 µM) compared to the same cells grown in 2D monolayer cultures in ambient air (Figure 4). Both spheroid and monolayer cultures were similarly sensitive to 48 h treatment with a high concentration of AP (25 µM) alone. We then tested the effect of AP treatment on the PLX4720-resistant phenotype of the 1205Lu spheroid and monolayer cultures. Co-treatment with both PLX4720 (25 µM) and AP (25 µM) led to a dramatic reduction in cell viability in the spheroid cultures unlike monolayer cultures that showed no further reduction in cell viability when compared to PLX4720 treatment alone (Figure 4). Thus, the increased resistance of the melanoma spheroid cultures to PLX4720 was eliminated with AP co-treatment.

## 4. Discussion

Melanoma is challenging to treat due to its genetic heterogeneity, and successful therapy requires targeting multiple molecular vulnerabilities. Our data show that melanoma tumor cells expressing mutant BRAF^V600E^ exhibit a high demand for polyamine growth factors and a greatly upregulated PTS. Utilizing the PTS for drug delivery, the AP compound attacks the melanoma cells via one of its key modes of survival. Indeed, we propose that polyamines are essential for the survival of melanomas. Polyamine levels are dramatically elevated in tumor cells compared to normal cells, often the result of oncogenic induction [11,20]. Previous studies have shown that the c-MYC and RAS can upregulate polyamine biosynthesis [9,10] and increase cellular uptake of polyamines by inducing PTS activity [12,13,14]. Although melanoma cells are notoriously replete with multiple oncogenic mutations, more than half of all melanoma tumors express a mutant BRAF protein [20]. Our data suggest that BRAF^V600E^ melanoma tumors have a greatly increased metabolic need for polyamines compared to normal cells. We have exploited the BRAF^V600E^-induced PTS activity in metastatic melanoma cells by targeting the PTS with AP.

Putrescine, spermidine, and spermine play key roles in cellular proliferation, signal transduction, gene expression, and autophagic states that contribute to tumor survival [32,33,34,35]. These endogenous polyamines and the polyamine-based AP compete to be imported into tumors via the PTS [21]. However, studies suggest that arylmethyl-polyamines similar to AP have enhanced cytotoxic potency via their multiple electrostatic interactions with DNA [36] and topoisomerase II [37]. Since AP selectively targets tumor cells with high polyamine transport rates, normal cells are significantly less sensitive to AP since they have low PTS activity [21]. Studies have shown that polyamine biosynthesis and cellular uptake are induced in hypoxic regions of tumors and in tumor spheroids [38]. Moreover, depletion of polyamines during hypoxia resulted in increased apoptosis [38], indicating that polyamines play an essential role in the ability of tumor cells to adapt to hypoxic stress and reactive oxygen species. Indeed, polyamines are known to exert anti-oxidant functions [39]. Although the melanoma spheroid cultures in this study were cultured with ambient air, it is well documented that the cells at the center of spheroids are hypoxic, thus modeling the heterogeneous 3D structure of in vivo melanoma tumors that often contain hypoxic regions [28,31]. Solid tumors contain poorly vascularized, hypoxic regions that contribute to tumor progression by activating a hypoxia stress response via hypoxia inducible factor-1α that promotes cell survival, tumor angiogenesis, and metastasis [40,41]. Studies have shown that hypoxic tumor cells and spheroid cultures are more resistant to chemotherapy including BRAF inhibitors [31,42,43]. Likewise, we have found that 3D cultures of 1205Lu melanoma cells grown as spheroids on NCPs are more resistant to PLX4720 treatment compared to 1205Lu cells grown in 2D monolayer culture in ambient air. Knowing that polyamine uptake is induced in hypoxic regions of tumor spheroids, we hypothesized that treatment with the PTS ligand AP would increase the sensitivity of 1205Lu spheroid cells to PLX4720. Indeed, the increased resistance of melanoma spheroids to PLX4720 was overcome with AP co-treatment. In contrast, AP co-treatment had no significant effect on the sensitivity of 2D monolayer cultures of 1205Lu cells to PLX4720.

Accumulating literature shows that treatment with a BRAF inhibitor such as PLX4720 enriches a slow-cycling cancer stem cell-like (CSC) subpopulation of melanoma cells that is characterized by stem cell markers such as Lysine-Specific Demethylase 5B (JARID1B) and spheroid formation [44,45]. It is thought that cancer stem cell populations exist in a hypoxic microenvironment [46,47,48]. Roesch et al. [45] have found that endogenous reactive oxygen species (ROS) levels are increased in slow cycling JARID1B^high^ melanoma cells as a result of increased mitochondrial respiration and oxidative phosphorylation. This high oxygen consumption contributes to hypoxic conditions that have been shown to favor the JARID1B^high^ slow-cycling CSC-like phenotype [44]. Using 1205Lu melanoma cells stably transduced with a JARID1B-promoter-green fluorescent protein (GFP)-reporter construct [44], we found that a short 2-day exposure to PLX4720 led to a 4-fold enrichment of JARID1B-driven GFP expressing 1205Lu melanoma cells grown as spheroids (data not shown). Our data show that PLX4720-resistant melanoma spheroids are made more sensitive to PLX4720 with AP co-treatment, and it is likely that AP is targeting CSC subpopulations that are enriched in the PLX4720-resistant melanoma spheroids.

Polyamines may also contribute to tumor survival by inducing an autophagic state [32,33,34,35]. For instance, spermidine has been shown to induce autophagy in multiple systems including yeast cells, *Caenorhabditis elegans*, *Drosophila melanogaster*, and human tumor cells [35,49] and to increase survival of pluripotent stem cells in culture [50]. Autophagy has recently emerged as a common survival process that tumors undergo when assaulted by chemotherapy and radiation [51]. It is induced by cellular stress such as nutrient deprivation, withdrawal of growth factors, and hypoxia [52]. In established tumors, autophagy is also a resistance mechanism to many therapeutic modalities including BRAF inhibitors [53]. Increased polyamine uptake provides a mechanism for BRAFi-resistant melanoma cells to acquire sufficient polyamines to undergo autophagy to survive treatment with BRAF inhibitors.

Previous clinical trials have tested the anti-tumor efficacy of the ODC inhibitor DFMO. However, treatment with DFMO alone demonstrated only moderate success in treating cancer patients [54]. Subsequent studies discovered that DFMO-inhibition of polyamine biosynthesis leads to upregulation of PTS activity with resulting increased uptake of polyamines from the diet and gut flora into the tumor cells [18]. Our findings show that DFMO induces PTS activity and increases AP sensitivity in melanoma cells that harbor a mutated BRAF protein. In summary, treatment with AP, with or without DFMO, offers an exciting potential as adjunct cancer therapy to overcome drug resistance in mutant BRAF^V600E^ melanoma.

## Figures and Tables

**Table 1:** Polyamine transport activity and sensitivity to AP in human melanoma cells with different BRAF mutational status cultured ± DFMO ^a^.

		− DFMO		+ DFMO	
Cell Line	BRAF Mutational Status	IC50 (µM AP)	PTS Activity ^b^ (cpm ^3^H Spd/µg Protein)	IC50 (µM AP)	PTS Activity ^b^ (cpm ^3^H Spd/µg Protein)
WM983B	V600E	2.6	487 ± 64	0.9	706 ± 78 *
WM3734	V600E	2.2	444 ± 46	1.2	654 ± 59 *
1205Lu	V600E	0.7	230 ± 22	0.6	299 ± 54
WM989	V600E	1.2	304 ± 28	1.0	348 ± 48
WM88	V600E	0.8	302 ± 18	0.2	343 ± 59
WM3451	WT	9.0	130 ± 21	5.1	157 ± 34
WM3743	WT	8.9	117 ± 23	10.9	110 ± 25
WM3211	WT	4.5	278 ± 58	5.2	251 ± 46

^a^ The mean values for half maximal inhibitory concentration (IC50) for AP and polyamine transport system (PTS) activity for human melanoma cells expressing mutant BRAF^V600E^ are compared with that for melanoma cells expressing wild type (WT) BRAF under conditions where cells were cultured without added DFMO or with 1 mM α-difluoromethylornithine (DFMO). ^b^ PTS activity expressed as counts per minute (CPM) ^3^H Spermidine (Spd)/µg protein ± standard deviation. PTS assays and cell viability assays were performed at least three times with each cell line. * *p* ≤ 0.001 when compared to the PTS activity in the absence of DFMO.

## Supplementary Materials

The following are available online at www.mdpi.com/2076-3271/6/1/3/s1, Figure S1: Greater PTS activity and increased sensitivity to AP in BRAF^V600E^ human melanoma cells compared to BRAF^WT^ cells.

## References

[1] Davies H., Bignell G.R., Cox C., Stephens P., Edkins S., Clegg S., Teague J., Woffendin H., Garnett M.J., Bottomley W. (2002). Mutations of the *BRAF* gene in human cancer. Nature.

[2] Solit D.B., Rosen N. (2011). Resistance to *BRAF* inhibition in melanomas. N. Engl. J. Med..

[3] Haq R., Fisher D.E. (2013). Targeting melanoma by small molecules: Challenges ahead. Pigment Cell Melanoma Res..

[4] Flaherty K.T., Puzanov I., Kim K.B., Ribas A., McArthur G.A., Sosman J.A., O’Dwyer P.J., Lee R.J., Grippo J.F., Nolop K. (2010). Inhibition of mutated, activated *BRAF* in metastatic melanoma. N. Engl. J. Med..

[5] Sosman J.A., Kim K.B., Schuchter L., Gonzalez R., Pavlick A.C., Weber J.S., McArthur G.A., Hutson T.E., Moschos S.J., Flaherty K.T. (2012). Survival in *BRAF* V600-mutant advanced melanoma treated with vemurafenib. N. Engl. J. Med..

[6] Hauschild A., Grob J.J., Demidov L.V., Jouary T., Gutzmer R., Millward M., Rutkowski P., Blank C.U., Miller W.H., Kaempgen E. (2012). Dabrafenib in *BRAF*-mutated metastatic melanoma: A multicentre, open-label, phase 3 randomised controlled trial. Lancet.

[7] Bello-Fernandez C., Packham G., Cleveland J.L. (1993). The ornithine decarboxylase gene is a transcriptional target of c-Myc. Proc. Natl. Acad. Sci. USA.

[8] Casero R.A., Marton L.J. (2007). Targeting polyamine metabolism and function in cancer and other hyperproliferative diseases. Nat. Rev. Drug Discov..

[9] Forshell T.P., Rimpi S., Nilsson J.A. (2010). Chemoprevention of B-cell lymphomas by inhibition of the Myc target spermidine synthase. Cancer Prev. Res..

[10] Origanti S., Shantz L.M. (2007). Ras transformation of RIE-1 cells activates cap-independent translation of ornithine decarboxylase: Regulation by the Raf/MEK/ERK and phosphatidylinositol 3-kinase pathways. Cancer Res..

[11] Poulin R., Casero R.A., Soulet D. (2012). Recent advances in the molecular biology of metazoan polyamine transport. Amino Acids.

[12] Bachrach U., Seiler N. (1981). Formation of acetylpolyamines and putrescine from spermidine by normal and transformed chick embryo fibroblasts. Cancer Res..

[13] Chang B.K., Libby P.R., Bergeron R.J., Porter C.W. (1988). Modulation of polyamine biosynthesis and transport by oncogene transfection. Biochem. Biophys. Res. Commun..

[14] Roy U.K., Rial N.S., Kachel K.L., Gerner E.W. (2008). Activated *K-*RAS increases polyamine uptake in human colon cancer cells through modulation of caveolar endocytosis. Mol. Carcinog..

[15] Pegg A.E. (1988). Polyamine metabolism and its importance in neoplastic growth as a target for chemotherapy. Cancer Res..

[16] Tabor C.W., Tabor H. (1984). Polyamines. Ann. Rev. Biochem..

[17] Pegg A.E. (1986). Recent advances in the biochemistry of polyamines in eukaryotes. Biochem. J..

[18] Gerner E.W., Meyskens F.L. (2004). Polyamines and cancer: Old molecules, new understanding. Nat. Rev. Cancer.

[19] Wallace H.M., Fraser A.V., Hughes A. (2003). A perspective of polyamine metabolism. Biochem. J..

[20] Seiler N., Delcros J.G., Moulinoux J.P. (1996). Polyamine transport in mammalian cells. An update. Int. J. Biochem. Cell Biol..

[21] Muth A., Kamel J., Kaur N., Shicora A.C., Ayene I.S., Gilmour S.K., Phanstiel O. (2013). Development of polyamine transport ligands with improved metabolic stability and selectivity against specific human cancers. J. Med. Chem..

[22] Phanstiel O., Kaur N., Delcros J.G. (2007). Structure-activity investigations of polyamine-anthracene conjugates and their uptake via the polyamine transporter. Amino Acids.

[23] Kramer D.L., Miller J.T., Bergeron R.J., Khomutov R., Khomutov A., Porter C.W. (1993). Regulation of polyamine transport by polyamines and polyamine analogs. J. Cell. Physiol..

[24] Nilsson J.A., Keller U.B., Baudino T.A., Yang C., Norton S., Old J.A., Nilsson L.M., Neale G., Kramer D.L., Porter C.W. (2005). Targeting ornithine decarboxylase in Myc-induced lymphomagenesis prevents tumor formation. Cancer Cell.

[25] Schayowitz A., Bertenshaw G., Jeffries E., Schatz T., Cotton J., Villanueva J., Herlyn M., Krepler C., Vultur A., Xu W. (2012). Functional profiling of live melanoma samples using a novel automated platform. PLoS ONE.

[26] Alhonen-Hongisto L., Seppanen P., Janne J. (1980). Intracellular putrescine and spermidine deprivation induces increased uptake of the natural polyamines and methylglyoxal bis(guanylhydrazone). Biochem. J..

[27] Obenauf A.C., Zou Y., Ji A.L., Vanharanta S., Shu W., Shi H., Kong X., Bosenberg M.C., Wiesner T., Rosen N. (2015). Therapy-induced tumour secretomes promote resistance and tumour progression. Nature.

[28] Yoshii Y., Waki A., Yoshida K., Kakezuka A., Kobayashi M., Namiki H., Kuroda Y., Kiyono Y., Yoshii H., Furukawa T. (2011). The use of nanoimprinted scaffolds as 3D culture models to facilitate spontaneous tumor cell migration and well-regulated spheroid formation. Biomaterials.

[29] Yamada K.M., Cukierman E. (2007). Modeling tissue morphogenesis and cancer in 3D. Cell.

[30] Haycock J.W. (2011). 3D cell culture: A review of current approaches and techniques. 3D Cell Culture: Methods and Protocols.

[31] Qin Y., Roszik J., Chattopadhyay C., Hashimoto Y., Liu C., Cooper Z.A., Wargo J.A., Hwu P., Ekmekcioglu S., Grimm E.A. (2016). Hypoxia-driven mechanism of vemurafenib resistance in melanoma. Mol. Cancer Ther..

[32] Cufi S., Vazquez-Martin A., Oliveras-Ferraros C., Martin-Castillo B., Vellon L., Menendez J.A. (2011). Autophagy positively regulates the CD44^+^ CD24^-/low^ breast cancer stem-like phenotype. Cell Cycle.

[33] Mirzoeva O.K., Hann B., Hom Y.K., Debnath J., Aftab D., Shokat K., Korn W.M. (2011). Autophagy suppression promotes apoptotic cell death in response to inhibition of the PI_3_K—mTOR pathway in pancreatic adenocarcinoma. J. Mol. Med..

[34] Morselli E., Galluzzi L., Kepp O., Marino G., Michaud M., Vitale I., Maiuri M.C., Kroemer G. (2011). Oncosuppressive functions of autophagy. Antioxid. Redox Signal..

[35] Morselli E., Marino G., Bennetzen M.V., Eisenberg T., Megalou E., Schroeder S., Cabrera S., Benit P., Rustin P., Criollo A. (2011). Spermidine and resveratrol induce autophagy by distinct pathways converging on the acetylproteome. J. Cell Biol..

[36] Dallavalle S., Giannini G., Alloatti D., Casati A., Marastoni E., Musso L., Merlini L., Morini G., Penco S., Pisano C. (2006). Synthesis and cytotoxic activity of polyamine analogues of camptothecin. J. Med. Chem..

[37] Wang H., Davis A., Yu S., Ahmed K. (2001). Response of cancer cells to molecular interruption of the CK2 signal. Mol. Cell Biochem..

[38] Svensson K.J., Welch J.E., Kucharzewska P., Bengtson P., Bjurberg M., Pahlman S., Ten Dam G.B., Persson L., Belting M. (2008). Hypoxia-mediated induction of the polyamine system provides opportunities for tumor growth inhibition by combined targeting of vascular endothelial growth factor and ornithine decarboxylase. Cancer Res..

[39] Mozdzan M., Szemraj J., Rysz J., Stolarek R.A., Nowak D. (2006). Anti-oxidant activity of spermine and spermidine re-evaluated with oxidizing systems involving iron and copper ions. Int. J. Biochem. Cell Biol..

[40] Pouyssegur J., Dayan F., Mazure N.M. (2006). Hypoxia signalling in cancer and approaches to enforce tumour regression. Nature.

[41] Keith B., Simon M.C. (2007). Hypoxia-inducible factors, stem cells, and cancer. Cell.

[42] O’Connell M.P., Marchbank K., Webster M.R., Valiga A.A., Kaur A., Vultur A., Li L., Herlyn M., Villanueva J., Liu Q. (2013). Hypoxia induces phenotypic plasticity and therapy resistance in melanoma via the tyrosine kinase receptors ROR1 and ROR2. Cancer Discov..

[43] Pucciarelli D., Lengger N., Takáčová M., Csaderova L., Bartosova M., Breiteneder H., Pastorekova S., Hafner C. (2016). Hypoxia increases the heterogeneity of melanoma cell populations and affects the response to vemurafenib. Mol. Med. Rep..

[44] Roesch A., Fukunaga-Kalabis M., Schmidt E.C., Zabierowski S.E., Brafford P.A., Vultur A., Basu D., Gimotty P., Vogt T., Herlyn M. (2010). A temporarily distinct subpopulation of slow-cycling melanoma cells is required for continuous tumor growth. Cell.

[45] Roesch A., Vultur A., Bogeski I., Wang H., Zimmermann K.M., Speicher D., Korbel C., Laschke M.W., Gimotty P.A., Philipp S.E. (2013). Overcoming intrinsic multidrug resistance in melanoma by blocking the mitochondrial respiratory chain of slow-cycling JARID1B^high^ cells. Cancer Cell.

[46] Schwab L.P., Peacock D.L., Majumdar D., Ingels J.F., Jensen L.C., Smith K.D., Cushing R.C., Seagroves T.N. (2012). Hypoxia inducible factor-1α promotes primary tumor growth and tumor-initiating cell activity in breast cancer. Breast Cancer Res..

[47] Mathieu J., Zhang Z., Zhou W., Wang A.J., Heddleston J.M., Pinna C.M., Hubaud A., Stadler B., Choi M., Bar M. (2011). HIF induces human embryonic stem cell markers in cancer cells. Cancer Res..

[48] Mohyeldin A., Garzon-Muvdi T., Quinones-Hinojosa A. (2010). Oxygen in stem cell biology: A critical component of the stem cell niche. Cell Stem Cell.

[49] Eisenberg T., Knauer H., Schauer A., Buttner S., Ruckenstuhl C., Carmona-Gutierrez D., Ring J., Schroeder S., Magnes C., Antonacci L. (2009). Induction of autophagy by spermidine promotes longevity. Nat. Cell Biol..

[50] Chen T., Shen L., Yu J., Wan H., Guo A., Chen J., Long Y., Zhao J., Pei G. (2011). Rapamycin and other longevity-promoting compounds enhance the generation of mouse induced pluripotent stem cells. Aging Cell.

[51] Strohecker A.M., White E. (2014). Targeting mitochondrial metabolism by inhibiting autophagy in *BRAF*-driven cancers. Cancer Discov..

[52] Kroemer G., Marino G., Levine B. (2010). Autophagy and the integrated stress response. Mol. Cell.

[53] Ma X.H., Piao S.F., Dey S., McAfee Q., Karakousis G., Villanueva J., Hart L.S., Levi S., Hu J., Zhang G. (2014). Targeting ER stress-induced autophagy overcomes *BRAF* inhibitor resistance in melanoma. J. Clin. Investig..

[54] Seiler N. (2003). Thirty years of polyamine-related approaches to cancer therapy. Retrospect and prospect. Part 1. Selective enzyme inhibitors. Curr. Drug Targets.

