# Supplementary material for: A Novel Polyamine-Targeted Therapy for BRAF Mutant Melanoma Tumors
Source: Med Sci (Basel). 2018 Jan 5;6(1):3. doi: 10.3390/medsci6010003 (PMC5872160; doi:10.3390/medsci6010003)
Supplement: Supplementary file 1 [file medsci-06-00003-s001.pdf]

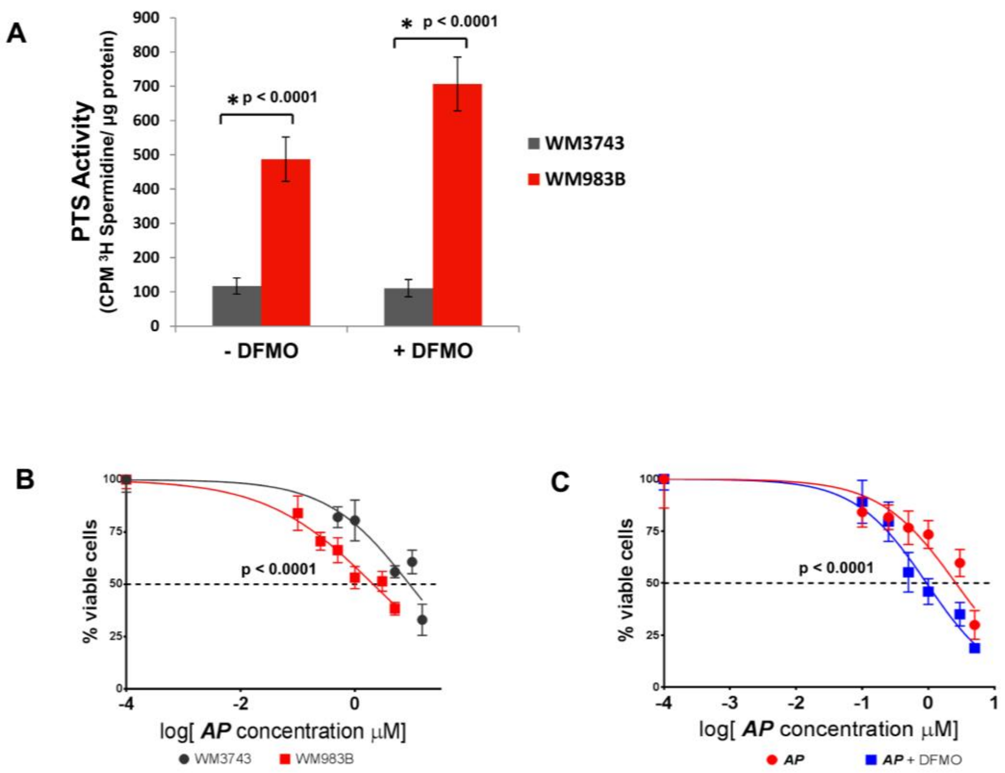

Figure S1. Greater PTS Activity and Increased Sensitivity to ***AP*** in $BRAF^{V600E}$ Human Melanoma Cells Compared to $BRAF^{WT}$ Cells. (**A**) $BRAF^{V600E}$ WM983B melanoma cells and $BRAF^{WT}$ WM3743 melanoma cells were cultured with and without 1 mM DFMO for 40 h and then pulsed with 0.5 µM ^{3}H-spermidine for 60 minutes at 37ºC. Cells were washed with cold PBS containing 50 µM spermidine, and cell lysates were assayed for CPM ^{3}H-spermidine per mg protein by scintillation counting. (**B**) WM983B and WM3743 melanoma cells were treated with increasing doses of ***AP***. After 72 h of culture, cell survival was determined via EZQuant™ Cell Quantifying assay. IC50 values were calculated by GraphPad Prism 6. (**C**) WM983B melanoma cells were treated with increasing doses of ***AP*** with or without 1 mM DFMO. After 72 h of culture, cell survival was determined via EZQuant™ Cell Quantifying assay. 72 h IC50 values were calculated by GraphPad Prism 6. Values are the mean of 5-6 samples ± SD.
